# Conserved genes regulating human sex differentiation, gametogenesis and fertilization

**DOI:** 10.1186/s12967-024-05162-2

**Published:** 2024-05-19

**Authors:** Khalid A. Fakhro, Johnny Awwad, Suma Garibova, Luis R. Saraiva, Matteo Avella

**Affiliations:** 1 Research Branch, Sidra Medicine, Doha, Qatar; 2 Weill Cornell Medicine, Doha, Qatar; 3 College of Health and Life Sciences, Hamad Bin Khalifa University, Doha, Qatar; 4 Reproductive Medicine Unit, Sidra Medicine, Doha, Qatar; 5 Obstetrics & Gynecology, American University of Beirut Medical Center, Beirut, Lebanon; 6 Vincent Memorial Obstetrics & Gynecology Service, The Massachusetts General Hospital, Boston, MA USA; 7 Department of Biomedical Sciences, Qatar University, Doha, Qatar

**Keywords:** Sperm, Oocyte, Egg, Genetics, Infertility, Fertility disorder, Transgenic, CRISPR/Cas, Knockout’

## Abstract

The study of the functional genome in mice and humans has been instrumental for describing the conserved molecular mechanisms regulating human reproductive biology, and for defining the etiologies of monogenic fertility disorders. Infertility is a reproductive disorder that includes various conditions affecting a couple’s ability to achieve a healthy pregnancy. Recent advances in next-generation sequencing and CRISPR/Cas-mediated genome editing technologies have facilitated the identification and characterization of genes and mechanisms that, if affected, lead to infertility. We report established genes that regulate conserved functions in fundamental reproductive processes (e.g., sex determination, gametogenesis, and fertilization). We only cover genes the deletion of which yields comparable fertility phenotypes in both rodents and humans. In the case of newly-discovered genes, we report the studies demonstrating shared cellular and fertility phenotypes resulting from loss-of-function mutations in both species. Finally, we introduce new model systems for the study of human reproductive biology and highlight the importance of studying human consanguineous populations to discover novel monogenic causes of infertility. The rapid and continuous screening and identification of putative genetic defects coupled with an efficient functional characterization in animal models can reveal novel mechanisms of gene function in human reproductive tissues.

## Background

Successful sexual reproduction requires the recognition and fusion of sperm and oocytes, which leads to the conception of new embryos. To produce healthy and functional gametes, the embryo must develop healthy gonads, which are populated by germ cells that differentiate at the onset of puberty. Molecular genetic studies have shown that thousands of genes play a role in regulating and preserving human reproduction. When one of the reproductive processes fails, the individual becomes unable to conceive. Infertility is defined as the inability to conceive after 6–12 months of unprotected sexual intercourse and affects 16.5–17.8% of couples globally [1].

Since the inception of human in vitro fertilization (IVF) [2], intracytoplasmic sperm injection (ICSI) [3], and the achievement of the first IVF pregnancies [4], extensive research has focused on optimizing in vitro insemination and embryo culture conditions in order to improve IVF outcomes. In addition, next-generation sequencing (NGS) technologies have also been adopted to decipher the causes of fertility phenotypes for faster and more precise diagnostics and treatments. Typical monogenic fertility disorders affect sperm number, motility, or morphology [5] and may lead to primary ovarian insufficiency (POI), abnormal zygote cleavage, and embryo development arrest [6]. These phenotypes are caused by loss-of-function variants affecting genes regulating mammalian reproduction [5, 6].

We searched PubMed and the Online Mendelian Inheritance in Man (OMIM) for established and newly identified genes that regulate conserved functions in fundamental mammalian reproductive processes (e.g., sex determination, gametogenesis, fertilization, and early embryo development). Of note, we included only genes for which studies on genome-edited rodents demonstrated conserved functions and comparable fertility phenotypes upon homozygous (or compound heterozygous) loss of function mutations (i.e., mutations leading to lack of protein expression) in humans [7–9]. For the established genes (genes with a well-documented conserved role in mouse and human), here we briefly describe their conserved role in mouse and human reproductive biology and reference only studies reporting the human fertility phenotypes. For the most recently identified genes, we describe shared cellular and fertility phenotypes resulting from loss-of-function mutations in both species.

We do not cover associative studies on genes for which no animal models have been generated. We do not include genes for which a null-mutant animal model has been generated, but no clear loss of function mutations has been found in humans (e.g., a homozygous putative deleterious missense variant without functional validation, or a heterozygous frameshift variant would not be considered as clear loss of function mutations). In addition, we do not include fertility phenotypes due to chromosomal structural aberrations, abnormal sex chromosome numbers (e.g., Y chromosome microdeletions, Klinefelter, XXY- XXXXY males), or genetic systemic disorders associated with infertility such as the Kartagener’s, fragile X, Noonan syndromes, myotonic dystrophy, sickle cell anaemia, and β-thalassemia.

### Genetic control of sex determination

Human sex determination occurs early in embryogenesis, and the embryo develops bipotential gonadal primordia, which through genetic regulation, can differentiate as either testes or ovaries [10]. Six weeks post-conception, the sex-determining region Y (*SRY*) gene activates Sry-related HMG box gene-9 (SOX9), which induces the expression of the Anti-Müllerian duct hormone (AMH), thus actively controlling testis development, Sertoli cell differentiation, and the general maleness of XY individuals [11]. Secreted by the Sertoli cells, AMH induces the degeneration of the Müllerian duct [12]. Sox9 also activates fibroblast growth factor-9 (*FGF9*), which represses the *WNT4* expression and the ovarian development [10]. In addition, SRY works in concert with the steroidogenic factor-1 (encoded by the Nuclear Receptor *NR5A1*) to maintain Sox9 expression [13].

**Fig. 1:**
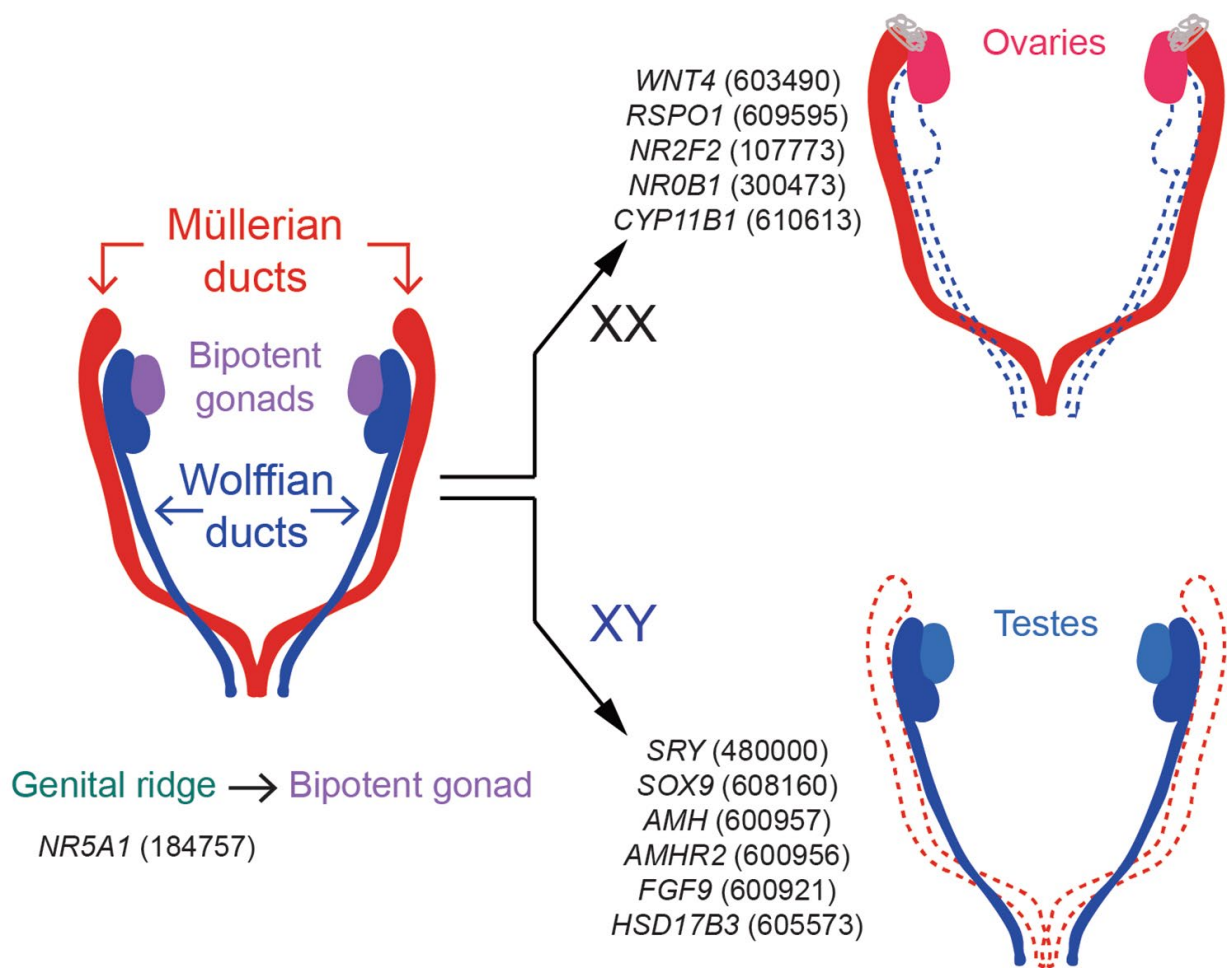
Genetics of human sex determination. Genes that mediate the differentiation of bipotent gonad or reproductive ducts and that are associated with comparable sex developmental disorders in mice and humans (OMIM gene ID).

Conversely, the Nuclear Receptor Subfamily 0 Group B Member-1 (NR0B1 or DAX1), antagonizes the function of SRY [14] while downregulating *NR5A1* expression [15] (Fig. 1). DNA variants affecting *SRY* and *SOX9* [16] leads to sex reversal in humans, and, on the other end, XX individuals carrying extra copies of either SRY or SOX9 develop as males [16]. Similarly, mutations in human *NR5A1* frequently lead to 46 XY disorders of sex development [17]. Consistent with its function, duplication of the X region containing *NR0B1* is associated with male-to-female sex reversal in XY individuals, and loss-of-function mutations in *NR0B1* are responsible for X-linked adrenal hypoplasia congenita, a disorder characterized by hypogonadotropic hypogonadism (Fig. 1) [18]. When gene expression favors the pro-ovarian Rspo1/Wnt4–β-catenin signaling pathway over Fgf9, a different set of genes takes over to regulate female sex determination (Fig. 1).

The Wingless-Type MMTV Integration Site Family, Member-4 (WNT4), is expressed in the genital ridge while still in its bipotential stage [11] and becomes undetectable in XY gonads. In contrast, it is maintained in XX as their gonads differentiate into ovaries. WNT4 expression is regulated by RSpondin-1 (RSPO1), a secreted activator protein that upregulates the canonical WNT/β-catenin signaling pathway to promote ovarian development while antagonizing testis formation. In humans, mutations in *WNT4* lead to 46 XX virilization, primary amenorrhea, uterine hypoplasia, and follicle depletion [19]. Similarly, XX individuals lacking RSPO1 show female-to-male sex reversal, and XY individuals with a duplicated Chromosome 1 region encompassing RSPO1 and WNT4 present male-to-female sex reversal [20]. These mechanisms are conserved across evolution, and gene deletion in transgenic mice leads to comparable sex reversal phenotypes and infertility. Of note, recent studies have shown that the elimination of the Wolffian ducts is an active process regulated by the Nuclear Receptor Subfamily-2-Group-F-Member-2 (*NR2F2*). This ligand-inducible transcription factor suppresses the mesenchyme-epithelium crosstalk, which is necessary to conserve the Wolffian ducts [21]. Gene deletion in mice leads to intersex individuals presenting female and male reproductive tracts [21]. In humans, loss-of-function mutations affecting *NR2F2* lead to testis [22] or ovotestis development [23] in XX individuals (Fig. 1).

Sex reversal can also manifest upon hormonal imbalance during embryo development. Mutations in *AMH* or its receptor *AMHR2* induce Persistent Müllerian Duct Syndrome and internal hermaphroditism [24–28]. In addition, deleterious variants in genes regulating hormonal biosynthesis are typically associated with sex-determination phenotypes. For example, deficiency in the enzymes regulating cortisol biosynthesis, such as Cytochrome P450 Family-11-Subfamily-B-Member-1 (CYP11B1) lead to congenital adrenal hyperplasia [29, 30]. Also, the Hydroxysteroid 17-beta Dehydrogenase-3 (*HSD17B3*) code for enzymes responsible for the biogenesis of testosterone and dihydrotestosterone. Lack of HSD17B3 leads to pseudohermaphroditism in 46 XY individuals, impaired maturation of Leydig cells, and under-masculinization in men and mice [31]. Similar fertility phenotypes are observed when *Amh, Ahmr2, Cyp11b1*, are deleted in transgenic mice.

While bipotent gonads commit to becoming ovaries or testes, gonadal somatic cells support the development of sex-specific germ cell lineages, precursors of eggs and sperm.

### Genes regulating germ cell physiology and meiosis in men

Over the past decade, advanced next-generation sequencing has unveiled the causes of numerous cases of male-factor infertility. Simultaneously, the generation of genome-edited mouse lines has shed light on the conserved functions of key mammalian genes, preserving male fertility in both mice and humans. Normal gametogenesis in mammalian males originates during early embryonic development from isomorphic primordial germ cells (PGCs) [32]. At this stage, the FA Complementation Group M (FANCM) gene is necessary to preserve genomic stability by regulating mammalian DNA replication and repair [33]. Deleterious variants in human *FANCM* lead to oligoasthenozoospermia [34] or Sertoli Cell-Only Syndrome, where only Sertoli cells outline the seminiferous tubules, with no sperm detectable [35], and *Fancm*-null male mice show reduced proliferation and loss of PGCs [36].

**Fig. 2:**
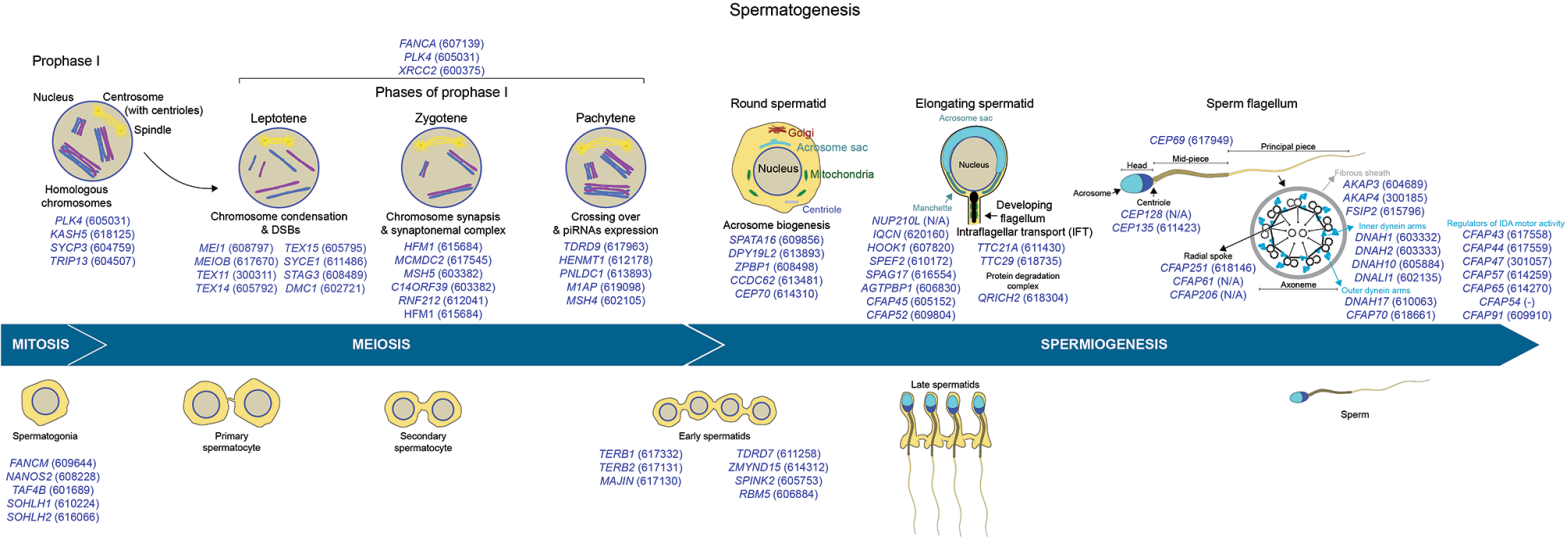
Genes regulating meiosis and spermiogenesis during spermatogenesis. Top, genes regulating different phases of Prophase I at meiosis or spermiogenesis stages (OMIM gene ID). Deletions of any of these genes present a comparable fertility phenotype in mice and humans (reported in OMIM or by case reports). Bottom, cellular differentiation stages of spermatogenesis

Male PGCs proliferate and migrate into the developing testis, where they will differentiate postnatally, into spermatogonia stem cells (SSCs) [37]. SSCs are unipotent cells that complement self-renewing with differentiating divisions to preserve the stem cell pool while maintaining adequate sperm production throughout the male reproductive lifespan [38]. Factors maintaining a balance between proliferating and differentiating SSCs help prevent the premature depletion of the SSC pool, while regulating controlled differentiation [38]. Human Nanos C2hc-Type Zinc Finger-2 (*NANOS2*) is necessary to prevent XY germ cells from prematurely entering meiosis, and one homozygous deleterious mutation has been found to segregate with Sertoli cell-only syndrome in humans [8]. Nanos2 role is conserved in mammals: Nanos2 deletion by Cas9 genome-editing in mice, pigs, goats, and cattle leads to germline-depleted testes and male infertility [39]. While Nanos2 prevents differentiation, the TATA-box Binding Protein Associated Factor-4b (TAF4B) controls the expression of genes promoting differentiation and self-renewal of SSCs, and *TAF4B* deletion results in nonobstructive azoospermia (NOA) or oligozoospermia in mice and men (Fig. 2) [40].

Spermatogonia undergo premeiotic DNA replication, differentiate into primary spermatocytes, and eventually enter prophase I of meiosis. The alignment and synapsis of the homologous chromosomes and genetic recombination occur during prophase I of meiosis. Meanwhile, the duplication of centrioles, the formation of DNA double-strand breaks (DSBs)(leptotene stage), the assembly and maintenance of the synaptonemal complex (zygotene stage), and the formation of crossing overs (pachytene stage) ensure the formation of genetically intact and fertile sperm (Fig. 2). The Polo-Like Kinase-4 (PLK4) controls centriole duplication, which is necessary for primary spermatocytes to undergo meiosis. One patient, carrier of a heterozygous deletion in the Ser/Thr kinase domain of PLK4, presented with infertility due to Sertoli cell-only syndrome, similar to mice heterozygous for a *Plk4* -null mutation [41].

The synaptonemal complex ensures the association between homologous chromosomes, while the cohesin complex mediates sister chromatid cohesion, and the telomeres adhere to and move on the nuclear envelope to regulate chromosome mobility and homologous pairing. Meiotic double-stranded break formation protein-1 (*MEI1*), Meiosis Specific with Ob-Fold (MEIOB), Testis Expressed-11 (TEX11), 14 (*TEX14*) and 15 (*TEX15*), help to induce the formation of DSBs, while contributing to the assembly of the synaptonemal complex and generation of crossovers between homologous chromosomes. Spermatocyte arrest is observed upon gene deletion of human *MEI1* [42] or *TEX11* [43–45], and in men carrying homozygous loss-of-function mutations in *MEIOB* [46–49]. In addition, depletion of TEX14 leads to Sertoli cell-only syndrome [8, 50], whereas deleterious variants in *TEX15* lead to NOA and crypto/oligozoospermia [45]. In addition, Minichromosome Maintenance Domain-Containing Protein-2 (*MCMDC2*) and Ring-Finger Protein-212 (*RNF212*) are necessary for the formation and maintenance of the synaptonemal complex and resolution of DSBs [8, 51, 52]. Moreover, three factors assemble in a complex that promotes telomere adhesion to the nuclear envelope, namely Telomere Repeat-Binding Bouquet Formation Protein-1 and − 2 (*TERB1*, *TERB2*), Membrane-Anchored Junction Protein (*MAJIN*) [52], and SAD1-and-UNC84-Domain-Containing-1 (*SUN1*) [53]. The TERB1-TERB2-MAJIN complex is necessary for mouse and human meiosis [54], and its disruption leads to NOA in men [52].

Screening more NOA patient genomes has confirmed the role of several other factors (that were already established as necessary for male meiosis in mice) in regulating human meiosis. Genes coding for meiosis-specific recombinases (DNA Meiotic Recombinase-1, *DMC1*) [55], proteins repairing DNA inter-strand crosslink and DSBs (*FANCA*) [56], transcription or post-transcriptional regulation such as Tudor-Domain Containing Protein-7 (*TDRD7*) [57] and *Zinc Finger Mynd-Containing Protein-15* (*ZMYND15*) [40], protein kinases such as *Serine Protease Inhibitor-Kazal-Type-2* (*SPINK2*) [58], or genes controlling meiotic progression (Meiosis-1-Associated-Protein, *M1AP*) [59] are necessary regulators of male meiosis in men and mice (Fig. 2).

Moreover, during male meiosis, the P-element–induced wimpy testis (PIWI)-interacting RNAs (piRNAs) (which are expressed in the pachytene spermatocytes) interact with PIWI proteins [60–62] (PIWIL1, or MIWI, PIWIL2, or MILI, and PIWIL4, or MIWI2) to preserve the germline genome against the mobilization of transposable elements [63]. The Hen Methyltransferase-1 (*HENMT1*) controls the 2’ O-methylation of piRNAs. HENMT1 deficiency results in piRNA instability and NOA [64]. In addition, adult meiotic and haploid germ cells undergo TE de-repression, resulting in the premature expression of haploid transcripts, increased DNA damage, and spermiogenesis arrest [65]. The Poly(A)-Specific RNAse-Like Domain Containing-1 (*PNLDC1*) regulates the processing of piRNAs by trimming the 3′ ends [66, 67]. Pnldc1 deletion in men and mice affected the expression of piRNA-processing proteins (e.g., PIWIL1, PIWIL4) and pachytene piRNAs in the testes, leading to NOA [66–68]. TDRD9 silences Line-1 (L1) retrotransposons in the male germ line [69], and loss of *TDRD9* leads to cryptozoospermia or azoospermia [69].

### Genetic control of female gametogenesis

DNA replication and recombination of oocyte meiosis occur in the fetal ovary, and the maturing oocytes arrest at the dictyate (diplotene) stage. At this stage, homologous chromosomes are held together in a bivalent configuration through crossover recombination between homologous chromosomes and cohesion between sister chromatids. In humans, such a configuration is maintained for decades until ovulation. At the resumption of oocyte meiosis, the completion of meiosis I coincides with the spindle formation, and the segregation of homologous chromosomes depends on the correct assembly of the spindle [70]. Tubulin-Beta-8 (*TUBB8*) is a necessary component of the mouse and human oocyte spindles. *TUBB8* DNA variants acting as dominant-negative led to infertility due to defective oocyte maturation, and an abnormal or completely-absent spindle [70, 71].

Spindle pole instability is another major cause of human fertility disorders, as it may lead to aneuploidy. Humans, bovine and porcine oocytes are depleted of acentriolar microtubule-organizing centers (aMTOCs), and the Nuclear Mitotic Apparatus Protein (NUMA)-mediated clustering of microtubule minus ends focuses the spindle poles [72]. In mice, the aMTOC-free oocytes present stable spindles owing to a spindle-stabilizing protein, the *KINESIN* Superfamily Protein-C1 (*KIFC1*), constitutively absent in human oocytes [72]. *Kifc1* deletion in mice leads to spindle instability, while exogenous KIFC1 injected in human oocytes rescues spindle instability [72].

At meiosis I, homologous recombination mediates accurate segregation of homologous chromosomes. *DMC1* regulates meiotic recombination by promoting homologous chromosome pairing and DNA strand transfer from nicked dsDNA to homologous ssDNA. *Dmc1-*null mice show aberrant oogenesis during fetal development and adult ovaries devoid of germ cells [55]. Similarly, in humans, homozygous deleterious mutation in DMC1 presented with primary infertility due to POI [55]. Psmc3-Interacting Protein (PSMC3IP) is another factor that facilitates meiotic recombination to promote DNA strand exchange at meiosis. Homozygous deleterious mutations in PSMC3IP have been shown to segregated with POI [73]. Similarly to humans, *Psmc3ip* null female mice present ovaries deprived of follicles and are infertile [73].

DNA repair by homologous recombination is a necessary step for the development of fertile oocytes. The repair of DNA double-strand breaks and stalled DNA replication forks during meiosis is mediated by two components of the mini-chromosome maintenance protein group, the Minichromosome Maintenance 8 Homologous Recombination Repair and Minichromosome Maintenance 9 Homologous Recombination Repair Factors (*MCM8*, *MCM9*). Several studies reported pathogenic variants in either MCM8 [74–77] or MCM9 [78, 79] segregating with POI. Comparably to humans, the deletion of mouse *Mcm8* or *Mcm9* leads to infertility due to ovaries lacking mature follicles [80]. During homologous recombination, the REC114 Meiotic Recombination Protein (REC114) mediates the formation of DNA DSBs in unsynapsed regions, a necessary step for the completion of synapsis. Lack of *Rec114* leads to NOA and POI in mice, due to defective DSB formation and aberrant homologous synapsis [81]; in women, *REC114* gene deletion leads to infertility due to supernumerary pronuclei formation at fertilization and early embryonic arrest [82].

### Genes that cause defective gametogenesis in men and women

Several genes regulate shared molecular pathways during male and female gametogenesis. These pathways include the pairing and recombination of homologous chromosomes, DNA repair, formation of crossovers, synaptonemal or cohesin complexes, or regulate gene expression during gametogenesis, independently from meiosis. Loss-of-function mutations affecting these genes may often result in NOA in men and POI in women [55, 73–80, 83–85]. Examples include MutS Homolog 4 and 5 (MSH4, and MSH5), X-Ray Repair Cross-Complementing-2 (XRCC2), Kash Domain-Containing Protein 5 (KASH5), DNA-Binding Protein-Synaptonemal Complex Protein-3 (*SYCP3*), Synaptonemal Complex Central Element Protein-1 (*SYCE1*), Thyroid Hormone Receptor Interactor-13 (TRIP13), Stromal Antigen 3 (STAG3), Spermatogenesis And Oogenesis Specific Basic Helix-Loop-Helix 1 and 2 (SOHLH1 and SOHLH2).

MSH4 mediates recombination and segregation of homologous chromosomes at meiosis in testes and ovaries, and gene deletion leads to POI [86] and NOA [87]. MSH5 regulates DNA mismatch repair and meiotic recombination, and deleterious variants also result in POI or NOA [88]. XRCC2 controls the homologous recombination DNA repair pathway during chromosomal fragmentation, translocations or deletions. While constitutional lack of *Xrcc2* leads to almost complete fetal or perinatal lethality, mice carrying a deleterious variant in *Xxrc2* present male infertility due to NOA and female infertility or severe female subfertility due to atrophic ovaries deprived of follicles [89]. Similarly, a deleterious variant in *XRCC2* has been shown to cause POI and NOA in humans [90]. During recombination, Helicase For Meiosis 1 (HFM1) regulates formation of crossover and complete synapsis of homologous chromosomes and its deletion leads to POI and NOA in mice [91] and POI in humans [92]. KASH5 regulates pairing of homologous chromosomes [93], and gene deletion leads to POI and NOA [8, 94].

SYCP3 also regulates homologous chromosome pairing and meiotic recombination. *SYCP3* DNA variants are associated with recurrent pregnancy loss, and *Sycp3* deletion in mice leads to oocyte aneuploidy [95]. Synaptonemal Complex Central Element Protein 1 (SYCE1) also connects homologous chromosomes during meiotic prophase I, and it is necessary for crossover formation. SYCE1 interfaces with Chromosome-14-Orf-39 (C14ORF39), a meiotic protein expressed in the central element of the synaptonemal complex. Deletion of *SYCE1* in two infertile sisters from a consanguineous family [96] or deleterious mutations in C14orf39 result in POI or NOA [51, 85, 96]. TRIP13 is a negative regulator of crucial elements of the synaptonemal complex, namely the HORMA proteins, HORMAD1 and HORMAD2 [97]. Deleterious missense mutations in *TRIP13* result in lower TRIP13 protein expression and to POI due to an aberrant intracellular accumulation of HORMAD2 mRNA and protein, which had a dominant effect, leading to oocyte meiotic arrest [98]; similarly, deletion of *Trip13* in mice leads to POI or NOA [99].

Cohesin is a chromosome-associated multi-subunit protein complex that preserves cohesion between replicated sister chromatids, and it is necessary for chromosome segregation and DNA repair. STAG3 is a subunit of the cohesin complex. Homozygous *STAG3* missense pathogenic variants associated with POI and NOA and *Stag3*-deficient mice show comparable phenotypes [83]. During gonad development, oocyte and spermatogonia differentiation are regulated by two transcription factors, SOHLH1 and 2. In males, Sohlh1 and 2 suppress genes that control SSCs maintenance and promote the expression of genes inducing spermatogonial differentiation. In females, they control oogenesis and folliculogenesis in the embryonic gonad. Both *Sohlh1*-null and *Sohlh2*-null mutant female mice are infertile due to severe lack of follicles [100], and *Sohlh1*-null and *Sohlh2*-null mutant males present spermatogonia that precociously enter meiosis [101]. In humans, lack of *SOHLH1* leads to ovarian dysgenesis [102].

### Spermiogenesis regulates the formation of fully differentiated sperm

Haploid spermatocytes undergo a series of key morphological changes, including acrosome biogenesis, DNA repackaging, head reshaping, and flagellum formation. These changes are orchestrated by several genes, whose mutations lead to fertility conditions related to sperm morphology and motility (e.g., globozoospermia, asthenozoospermia, and asthenoteratozoospermia) [103].

During acrosome biogenesis, small Golgi-derived proacrosomic vesicles amass and merge into a single spherical acrosomic vesicle that connects to the nucleus [103]. Deleterious mutations in genes regulating acrosome biogenesis, such as Spermatogenesis Associated-16 (*SPATA16*), Dpy-19 Like-2 (*DPY19L2*), Zona Pellucida Binding Protein-1 (*ZPBP1*) and the Coiled-Coil Domain-Containing-62 (*CCDC62*), typically lead to globozoospermia, defined by sperm with a round-shaped head, and an atrophied, misplaced, or virtually absent acrosome, as shown by studies in consanguineous and non-consanguineous human populations [104–106]. Meanwhile, the sperm nucleus is efficiently compacted to facilitate the delivery of the paternal genome to the egg. Nucleoporin − 210-Like (*NUP210L*) controls nuclear trafficking at spermiogenesis, and loss-of-function variants in human *NUP210L* in one infertile consanguineous man resulted in low sperm count, poor motility, and large-headed sperm presenting uncondensed nuclear DNA [107].

The structural reshaping of the spermatocyte head is regulated by a transient microtubular structure defined as the *manchette*, which mediates the condensation and elongation of the sperm head and the development of the flagellum [108]. During the manchette assembly, the IQ Motif-Containing-N (*IQCN*) regulates microtubule nucleation through calmodulin and calmodulin-related binding proteins [109]. The Hook Microtubule-Tethering Protein-1 (*HOOK1*) mediates the formation of the manchette and the intracellular transport of proteins [110]. Sperm Associated Antigen-17 (*SPAG17*) is a component of the sperm manchette and axoneme [111]. The ATP/GTP Binding Protein 1 (AGTPBP1) regulates the polyglutamation of tubulin during spermiogenesis. AGTPBP1 is expressed in the mouse and human manchette, and its absence leads to teratozoospermia and infertility in mice and humans [112]. The Cilia And Flagella Associated Protein-52 (*CFAP52*) codes for an inner microtubule protein necessary for the ciliary or flagellar beating. CFAP52 works with CFAP45 and axonemal dynein subunit *DNAH11* and localizes to the spermatid manchette and the sperm tail. In addition, CFAP69 regulates head and flagellum development [113], whereas the Centrosomal Protein 70 (CEP70) mediates flagellar formation and acrosome biogenesis [114, 115]. Deleterious variants affecting the manchette development lead to aberrant acrosomal morphology [109], decaudated heads or headless tails [110], severely reduced sperm motility [116], or asthenozoospermia [117].

The flagellum is another essential structural component of mature sperm as it confers progressive and hyperactive motility, both necessary for fertilization. Numerous factors define the flagellum formation and elongation. The development and function of each of these factors are regulated by individual genes that, if deleted, lead to asthenozoospermia and multiple morphological abnormalities of the flagella (MMAF; defined by short, coiled, irregular, or absent sperm tails). The centrosomes are organelles that play a dual role, before and after fertilization. Before fertilization, centrosomes link the head and tail and regulate sperm flagellar beating; after fertilization, centrosomes mediate the formation of the zygote cytoskeleton [118]. The Centrosomal Protein-128 and 135 (*CEP128; CEP135*) mediate centriole biogenesis [119, 120]. The Intraflagellar transport (IFT) complex controls protein transport along the developing flagellum, and the Tetratricopeptide Repeat Domain-21a (*TTC21A*) [121] and − 29 (*TTC29*) are key regulators of the IFTs [122, 123]. The fibrous sheath (FS) provides the sperm with proper structure, flexibility, and regulation of motility through the activity of A-Kinase Anchoring Protein-3 and − 4 (*AKAP3*, *AKAP4*), and the Fibrous Sheath Interacting Protein-2 (*FSIP2*) [124, 125]. The axoneme of the flagellum is defined by a “9 + 2” structure consisting of a central pair of two singlet microtubules surrounded by nine doublet microtubules. It confers motility to the sperm through the Inner and Outer Dynein Arms (IDAs, ODAs) motor activity [126]. Sperm Flagellar Protein-2 (*SPEF2*) is necessary to develop the axoneme [126, 127].

In addition, several axonemal dynein proteins, including *DNAH1*, *DNAH2*, *DNAH6*, *DNAH10*, *DNAH17*, and *DNALI1* [128–138], Cilia And Flagella Associated Proteins (*CFAP43, CFAP44, CFAP47, CFAP54, CFAP57, CFAP65, CFAP70*) are main constituents of the IDAs and ODAs and loss of function variants lead to defective spermiogenesis and male infertility [139–145]. Finally, the radial spoke is a multiprotein complex (CFAP61, CFAP91, CFAP206, CFAP251) [146–149] serving as a mechanochemical transducer between the central and peripheral pair microtubule doublets while controlling flagellar beating. Also, during flagellar formation, the ubiquitin-proteasome pathway eliminates abnormal proteins, organelles, and sperm cells. Glutamine Rich-2 (*QRICH2*) stabilizes the expression of proteins necessary for flagellar development by suppressing the ubiquitination-dependent degradation of these proteins [150, 151]. Indeed, the exact splicing of pre-mRNAs from genes regulating spermatid head structuring and acrosome and tail biogenesis is imperative for proper spermiogenesis. The RNA-Binding Motif Protein-5 (*RBM5*) is a key component of the spliceosome A complex, and deleterious mutations lead to defective spermatid differentiation and NOA [152].

Spermiogenesis finally results in the development of mature spermatozoa that are released from the seminiferous tubules into the epididymis to undergo post-testicular maturation before becoming competent for natural fertilization. Different molecular changes in the growing ovarian follicle and developing oocyte are necessary to develop a fertile egg.

### Follicular maturation and formation of the zona pellucida

The control of ovarian function, somatic granulosa cells’ fate, and oocyte’s developmental competence are under the concerted action of the Growth/Differentiation Factor-9 (*GDF9*) and the Bone Morphogenetic Protein-15 (*BMP15*). GDF9 and BMP15 belong to the TGF-beta of ligands that activate SMAD family transcription factors. Homozygous frameshift deletion in *GDF9* leads to POI and infertility in women and mice [153]. BMP15 can heterodimerize with GDF9 to promote oocyte maturation and follicular development by regulating gene expression in granulosa cells.

Genome integrity and appropriate control of mRNA metabolism are also necessary during oocyte maturation. The transcription factor p63 (TP63) maintains the female germline genome intact during oogenesis. Heterozygous *TP63* gene deletion leads to the formation of an aberrantly activated mutant TP63 tetramer, which acts in a dominant negative fashion by increasing the expression of apoptosis-inducing factors, leading to cell apoptosis in the ovary and POI in mice and humans [154]. Pat1-Homolog-2 (*PATL2*) regulates transcription and translation during oogenesis, and loss of *PATL2* leads to infertility in women due to oocyte maturation arrest [155]. Deletion of *Patl2* in mice affects the expression of key transcripts during oocyte maturation, leading to a decreased number of ovulated MII oocytes and defective early embryo development [156].

**Fig. 3:**
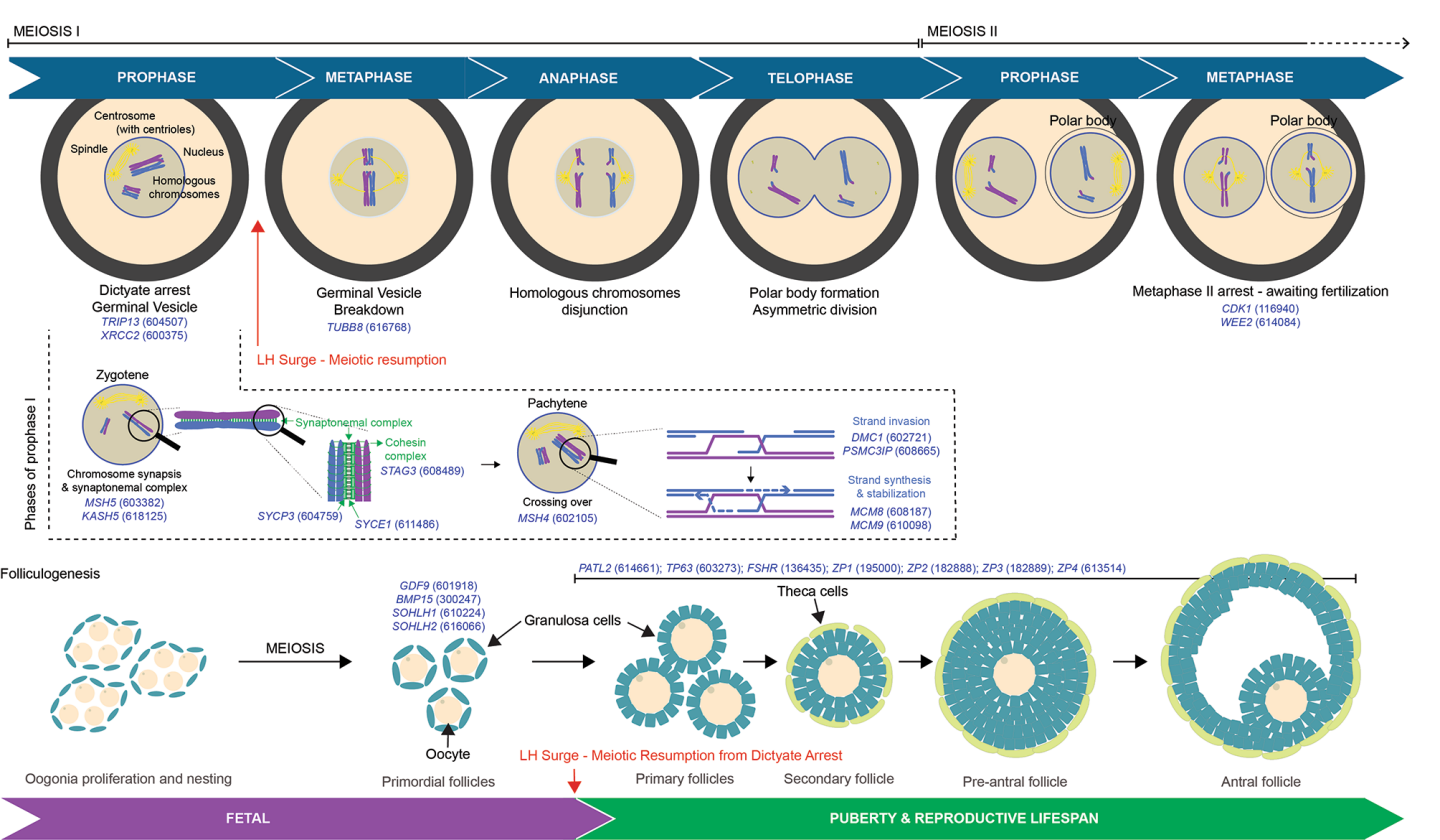
Genetics of oogenesis and follicular development. Top, genes regulating different stages of meiosis I and II, or phases at meiosis I (gene OMIM ID). Deletions of these genes present a comparable fertility phenotype in mice and humans. Bottom, stages of folliculogenesis at pre- or post-birth and genes regulating oocyte maturation (OMIM gene ID). Deletions of these genes present a comparable female (or female and male, for genes regulating meiosis) fertility phenotype in mice and humans (reported in OMIM or by case reports)

In addition, aberrant ovarian development leads to ovarian dysgenesis likely resulting in atrophic ovaries and absence of folliculogenesis. Indeed, lack of Follicle Stimulating Hormone Receptor (FSHR) leads to atrophic ovaries, loss of folliculogenesis and defective ovulation in mice [157] and ovarian dysgenesis in humans [158].

Human oocytes are surrounded by the extracellular zona pellucida (ZP), composed of 4 glycoproteins, ZP1-4 [7]. Mutations of the *ZP* genes affect the zona structure; homozygous frameshift or compound heterozygous variants affecting *ZP1* result in mutant ZP1 that sequesters ZP3 during zona biogenesis [159, 160] or prevent the establishment of filament crosslinking in the matrix, which typically preserves the structural stability of the zona, leading to zona absence and infertility [161–163]. In addition, women or female mice with heterozygous nonsense mutations in ZP2 and frameshift mutations in ZP3 had no zona formation and primary infertility [164]. Moreover, deleterious missense mutations in ZP3 prevent proper interaction with ZP2, leading to empty follicle syndrome due to the absence of zona formation [165]. A structurally intact zona surrounding a genetically-intact MII oocyte is the prerequisite for successful fertilization (Fig. 3).

### Fertilization and early embryo development

**Fig. 4:**
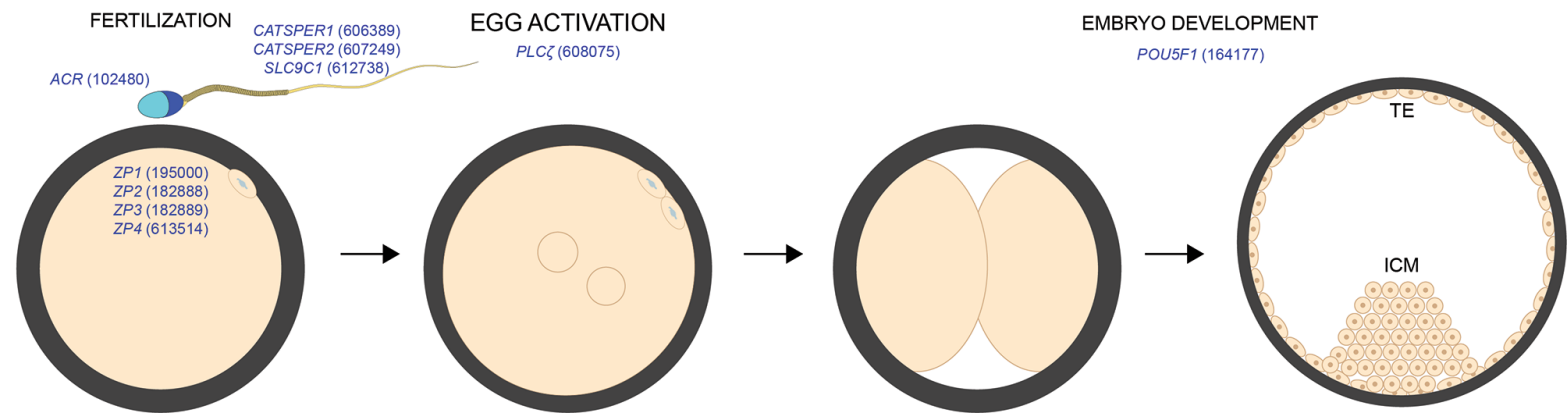
Genetics of fertilization and preimplantation development. Genes coding for proteins mediating sperm physiology, gamete interaction, and early embryo development (gene OMIM ID). Deletions of these genes present comparable or dissimilar fertility phenotypes in mice vs. humans

Following asymmetric cytokinesis, oocytes enter metaphase II (MII) and complete meiosis II only after fusion with the fertilizing sperm. Inhibition of Cyclin-Dependent Kinase-1 (*CDK1*) induces meiosis II completion after gamete fusion. The inhibition of CDK1 is regulated by the WEE2-Oocyte Meiosis-Inhibiting Kinase (*WEE2*), which is a regulator of cell cycle progression (Fig. 3). Women with deleterious variants in *WEE2* show oocyte maturation defects [166] or recurrent fertilization failure (due to aberrant oocyte maturation) [167]. For successful fertilization, sperm must swim toward the unfertilized egg, undergo acrosome exocytosis, and bind and cross the extracellular zona. After gamete adhesion and fusion, egg activation allows the resumption of meiosis and the beginning of preimplantation embryo development [7] (Fig. 4). The Solute Carrier Family 9 Member-C1 (*SLC9C1*) is a sodium/proton exchanger that controls sperm motility through soluble adenylyl cyclase in men and mice [168]. In addition, the Potassium Channel, Subfamily U, Member-1 (*KCNU1*), mediates sperm membrane hyperpolarization and acrosome exocytosis, and men from consanguineous families and mice lacking KCNU1 are infertile due to impaired acrosome exocytosis and zona penetration [169].

Also, Acrosin, a trypsin-like serine protease in the sperm acrosome, mediates sperm passage through the zona, and gene deletion in consanguineous men and hamsters leads to infertility [170, 171]. Before crossing the zona, sperm bind to ZP2 [172]. Two homozygous ZP2 variants in consanguineous women led to zonae that could not support sperm binding, and women were infertile [173]. To cross the zona, sperm must also acquire hyperactive motility, a vigorous non-linear swimming pattern, which, in mice, is mediated by CatSper, a sperm-flagellar specific and Ca^2+^-selective channel [174]. CatSper is composed of nine known different proteins coded by individual genes. Loss of *CATSPER1* or *CATSPER2* leads to infertility in men due to reduced sperm count [175] or asthenoteratozoospermia [176].

After crossing the zona, acrosome-reacted sperm adhere to the oolemma through the direct interaction between the sperm membrane ligand IZUMO1 and its oocyte receptor, JUNO [177]. After adhesion, gametes fuse, and the sperm protein Phospholipase C-Zeta-1 (PLCζ1) mediates egg activation through Ca^2+^ signaling. Consanguineous men lacking PLCζ1 present normal sperm parameters, yet they cannot fertilize eggs due to a defective oocyte activation [178, 179]. In addition, deleterious mutations in *PLCζ1* induce a mislocalized and decreased expression of PLCζ1 in the sperm head, which results in infertility [180]. Also, men with homozygous or compound heterozygous pathogenic variants in Actin-Like7a (*ACTL7A*) and 9 (*ACTL9*) present sperm lacking PLCζ expression in the head, which leads to failed oocyte activation [181, 182]. Contrarily to humans, transgenic male mice lacking PLCζ1 are subfertile, as their sperm often (though not always) fail to activate the MII oocytes [183].

After fertilization, preimplantation embryo development is regulated by preserving a precise balance between cell pluripotency and cell differentiation, which is necessary for successful implantation (Fig. 4). In the blastocyst, the Caudal Type Homeobox-2 (*CDX2*)-expressing trophectoderm and the pluripotent inner cell mass (ICM) define two distinct lineage specifications. In humans, Pou Domain-Class-5-Transcription Factor-1 (*POU5F1*) transcripts are detected at the four-to-eight-cell stage, yet the OCT4 protein is detectable at the eight-cell stage [184]. Genome editing studies have reported that deletion of *POU5F1* by CRISPR/Cas9 in human zygotes resulted in developmental defects before blastocyst formation [185]. Loss of *POU5F1* in human embryos leads to failure to complete blastocyst formation and embryonic lethality [185].

## Conclusions

Almost half a century after the first baby was conceived through IVF [2], our understanding of the genetics and molecular biology underlying fertility disorders has remarkably increased. Genetic reports on infertile individuals from populations with high consanguinity rates have successfully identified more infertility-causing mutations. Besides, CRISPR/Cas-genome editing tools have facilitated the functional characterization of genes regulating mammalian reproduction. Thus, the increased available numbers of known genes and variants causing fertility phenotypes allow fertility specialists to treat patients with personalized therapies based on their genetics. However, despite the increased understanding of the functions of individual reproductive proteins, a few key questions still need to be answered.

*Can deleterious heterozygous variants in multiple genes affect the fertility of one individual?* Recent studies in mice show how even deleterious heterozygous variants affecting distinct but functionally related genes may lead to reproductive phenotypes such as male infertility due to MMAF [186]. These discoveries raise the hypothesis that some idiopathic male infertility cases could be explained by heterozygous deleterious variants affecting multiple loci within the same intracellular pathways.

*Is the mouse the best organism to model human reproductive disorders?* Combining genome sequencing of infertile consanguineous individuals with the deletion of candidate genes in transgenic mice can be an effective strategy for studying the genetics of infertility. However, deleting conserved genes in different mammalian species may lead to differences in the severity of fertility phenotypes or the sex affected. For example, the genetic ablation of Acrosin results in no fertility phenotype in mice, subfertility in rats, or complete infertility in hamsters and humans [170, 171]. Moreover, because Piwi genes regulate spermatogenesis in mice, their role in preserving women’s fertility has been neglected. However, in hamsters, Piwi genes control oogenesis and early embryo development, and deleting genes regulating the Piwi-interacting RNAs pathway in hamsters leads to male and female infertility [187, 188]. Interestingly, the expression pattern of Piwi genes in humans is highly similar to hamsters. Therefore, the hamster represents another promising model for studying human infertility.

*Does inbreeding increase the incidence of monogenic forms of infertility?* From a public health perspective, it is still unclear whether consanguineous populations experience a higher incidence of monogenic forms of infertility [189]. Several studies report that inbreeding increases the relative risk for monogenic recessive disorders; thus, it is reasonable to hypothesize that inbreeding augments the risk of monogenic infertility.

To address these questions, future research should continue combining NGS of infertile patients with mammalian models of reproductive disorders to expand the knowledge on the genetics of infertility. In addition, genomic data from inbred populations will help determine whether high consanguinity rates lead to increased primary infertility cases.

## Data Availability

The data underlying this article are available in the article.

## Abbreviations

NOA: nonobstructive azoospermia
POI: primary ovarian insufficiency
MMAF: multiple morphological abnormalities of the flagella
NGS: Next generation sequencing
ICSI: Intracytoplasmic sperm injection
IVF: In vitro fertilization
